# QT monitoring in chemotherapy

**DOI:** 10.3389/fcvm.2026.1748382

**Published:** 2026-05-19

**Authors:** Peter Y. Kim, Keila Carolina Ostos Mendoza, Jung Hyun Kim, Silvia Fernanda López Moreno, Bernardo Casso-Chapa, Noah I. Beinart, Angelica Paniagua-Bojorges, Andrea Mariana Ruesta Carrion, Stefania Assunto Lenz, Syed Wamique Yusuf, Anita Deswal, Nicolas L. Palaskas, Efstratios Koutroumpakis, Jun-ichi Abe, Michael S. Ewer

**Affiliations:** Division of Internal Medicine, Department of Cardiology, The University of Texas MD Anderson Cancer Center, Houston, TX, United States

**Keywords:** cardiac monitoriing, cardio-oncology, chemotherpay-induced cardiotoxicity, QT interval assessment, QT prolongation

## Abstract

This review provides a comprehensive overview of cardiac electrophysiology principles as they apply to oncology care, with a focus on QT interval assessment and monitoring. Accurate measurement of the QT interval and appropriate heart rate correction—particularly using the tangent method and Fridericia formula—are essential for evaluating arrhythmic risk in cancer patients, who are especially vulnerable due to the cardiotoxic potential of many chemotherapeutic agents. We highlight key risk factors for QT prolongation, including electrolyte imbalances, underlying cardiac conditions, and concurrent use of QT-prolonging medications. Current cardiac monitoring protocols during chemotherapy are examined, with specific reference to clinical practices at The University of Texas MD Anderson Cancer Center. These practices underscore the importance of structured surveillance and timely intervention. Effective management requires a multi-pronged approach involving standardized measurement techniques, proactive risk stratification, and patient education. Counseling patients on symptoms such as palpitations, dizziness, or syncope facilitates early detection and evaluation. Given the expanding list of QT-prolonging agents, clinicians are encouraged to use resources like Torsades.org for up-to-date drug information. In the absence of formal guidelines for certain agents, institution-specific protocols defining thresholds for treatment initiation, dose adjustment, and monitoring frequency are critical for ensuring consistency, safety, and evidence-based decision-making in cardio-oncology.

## 1 Introduction

This review aims to provide a comprehensive overview of critical concepts and practices related to cardiac electrophysiology in oncology care. Specifically, it will delve into the methodology for accurately measuring the QT interval and applying heart rate correction techniques, which are essential for assessing cardiac risk. A key focus will be the identification of risk factors contributing to QT interval prolongation within the cancer patient population—a group particularly vulnerable due to the cardiotoxic potential of various chemotherapeutic agents.

In addition, the session will examine the current guidelines for cardiac monitoring during chemotherapy, highlighting how these protocols are operationalized at The University of Texas MD Anderson Cancer Center. As of 2023, MD Anderson conducted approximately 88,500 electrocardiograms (ECGs) annually. Notably, nearly 3,900 of these ECGs revealed a corrected QT interval (QTc) exceeding 500 milliseconds—a threshold associated with heightened risk for ventricular arrhythmias. These findings underscore the importance of vigilant cardiac surveillance and timely intervention in oncology patients undergoing treatment.

## 2 Case report #1, torsades de pointes

The first clinical case presented is a 66 year old female diagnosed with acute myelogenous leukemia, who went through treatment with hydroxyurea followed by idarubicin and cytarabine. Her treatment course was complicated by respiratory failure and neutropenic fever. The patient presented symptoms of gait instability, dizziness, and falls, for which she was admitted for neurologic and cardiac evaluations. The patient was taking the following medications at the time of her admission: promethazine, ondansetron, senokot, pantoprazole, risperidone, temazepam, tramadol, estrogen, levothyroxine, moxifloxacin, ciprofloxacin, voriconazole, valacyclovir, ipratropium, cetirizine, and levalbuterol.

Key laboratory findings revealed significant cytopenias and electrolyte abnormalities. The white blood cell (WBC) count was markedly reduced at 1.1 × 10⁹/L, hemoglobin measured 9.6 g/dL, and platelet count was 76 × 10⁹/L, indicating pancytopenia. Electrolyte analysis showed hyponatremia with a sodium level of 132 mmol/L and hypokalemia with a potassium level of 3.3 mmol/L. Magnesium was slightly below normal at 1.8 mg/dL, while calcium was measured at 8.4 mg/dL. Phosphorus was within normal limits at 3.8 mg/dL.

Neuroimaging via magnetic resonance imaging (MRI) of the head demonstrated no evidence of acute infarction or leptomeningeal involvement. Cardiac evaluation included a transthoracic echocardiogram, which revealed a left ventricular ejection fraction (LVEF) of 35% which was a significant decline from her previously documented LVEF of 55%–60%. To further investigate the etiology of this cardiac dysfunction, she underwent cardiac catheterization. The procedure did not identify any significant obstructive coronary artery lesions that could account for the observed reduction in LVEF.

**Figure 1:**
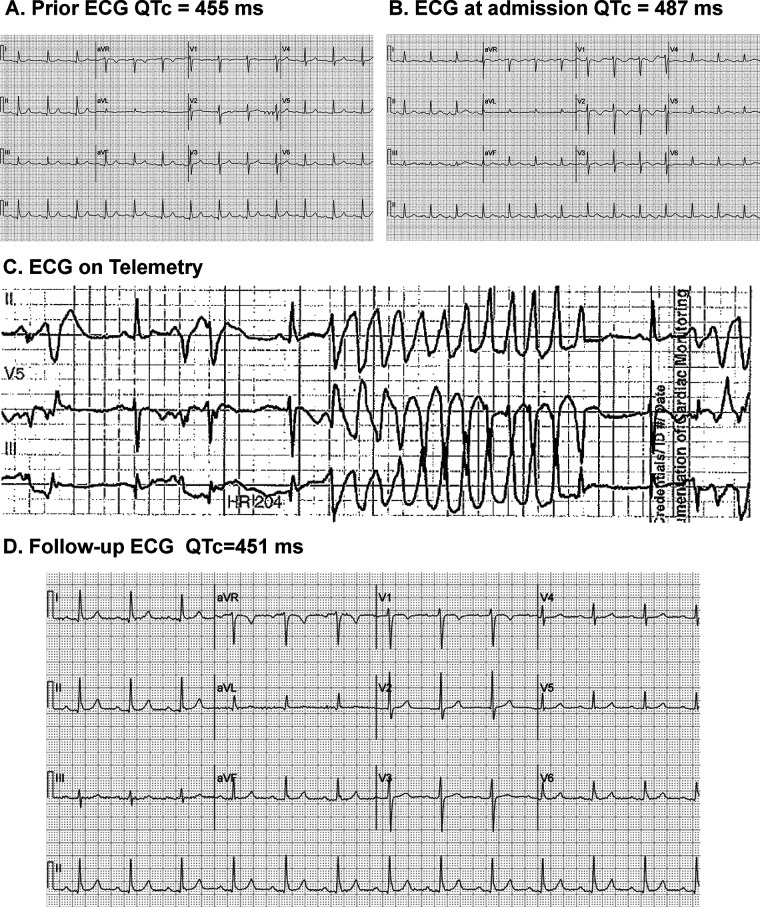
Serial electrocardiograms demonstrating QTc changes and arrhythmia. **(A)** Prior ECG: Baseline tracing showing sinus rhythm with a corrected QT interval (QTc) of 455 ms. **(B)** ECG at Admission: Sinus rhythm with QTc prolongation to 487 ms. **(C)** Telemetry Recording: Episode of polymorphic ventricular tachycardia consistent with torsades de pointes, occurring during hospitalization. **(D)** Follow-up ECG: Sinus rhythm restored with QTc shortened to 451 ms after clinical intervention.

While her past ECGs had been normal (Figure 1A), the ECG she presented at admission showed some biphasic inverted T waves and a QTC of 487 ms, which was around 30 ms longer than her past ECGs (Figure 1B). When she was admitted to the hospital she was placed on cardiac monitoring and telemetry. While she was on telemetry, she started showing a lot of PVCs and premature complexes, as well as short runs of non-sustained VT in twisting patterns consistent with Torsades de pointes (Figure 1C).

**Figure 2:**
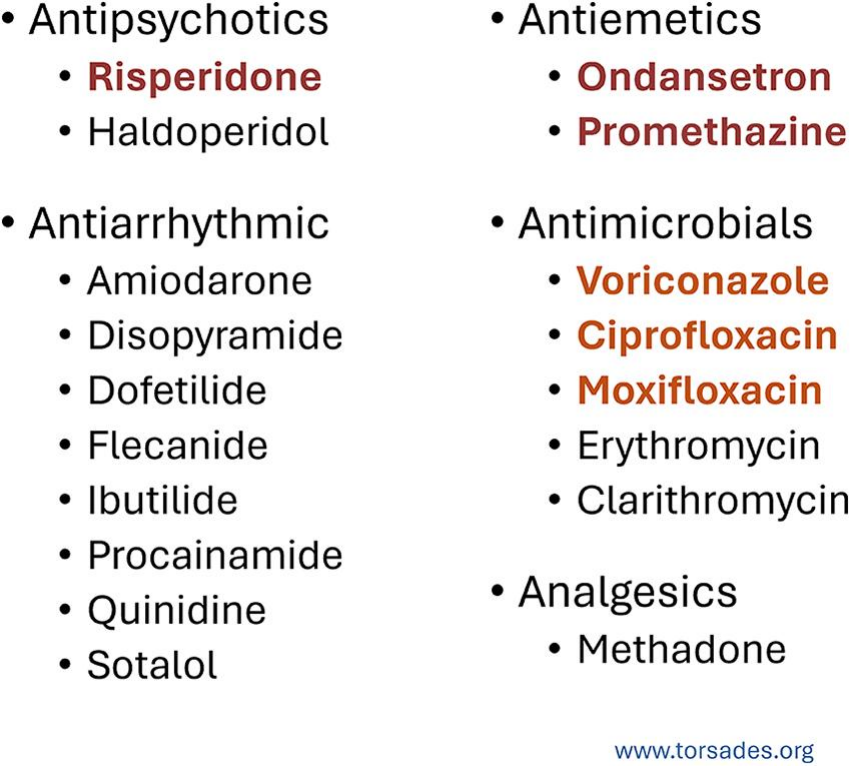
Drug classes associated with QT interval prolongation and risk of torsades de pointes. This figure lists common medications that can prolong the QT interval and increase the risk of torsades de pointes, grouped by therapeutic class. These include antipsychotics (e.g., risperidone, haloperidol), antiemetics (ondansetron, promethazine), antiarrhythmics (such as amiodarone, dofetilide, sotalol), antimicrobials (including voriconazole, ciprofloxacin, moxifloxacin, erythromycin, and clarithromycin), and analgesics (methadone). Drugs highlighted in brown represent those with a higher or more frequent association with QT prolongation.

A variety of medications are known to cause QT interval prolongation, predisposing patients to arrhythmias such as Torsades de pointes. Drug classes commonly implicated include antipsychotics, antiarrhythmics, antiemetics, antimicrobials, and certain analgesics (1, 2). In cardio-oncology, awareness of these agents is particularly critical because many chemotherapeutic and supportive are drugs, such as tyrosine kinase inhibitors (nilotinib, sunitinib, crizotinib) (3), arsenic trioxide (4), and antiemetics like ondansetron (5), all exhibit QT-prolonging potential (Figure 2).

In this case, the patient was taking several medications that are associated with QT prolongation including risperidone, ondansetron, promethazine, voriconazole, and moxifloxacin, many of which are commonly prescribed to inpatients in a hospitalized setting. After the patient received treatment and electrolyte correction, the patient's repeated ECG demonstrated normalization of the QTc interval to 451 ms and resolution of T-wave inversions, consistent with recovery from transient induced QT prolongation.

## 3 From QT prolongation to torsades de pointes

Prolongation of the QT interval can evolve into Torsades de Pointes, a distinct form of polymorphic ventricular tachycardia characterized by a “short-long-short” initiating sequence (6). Torsades de Pointes is particularly dangerous as it may rapidly degenerate into ventricular fibrillation, cardiac arrest, or asystole if untreated (7).

This underscores that the cornerstone of effective management in patients at risk for QT interval prolongation involves two critical strategies: prompt correction of electrolyte disturbances and the judicious discontinuation of medications known to prolong the QT interval. Electrolyte imbalances particularly drug-induced hypokalemia and hypomagnesemia are well-established contributors to delayed cardiac repolarization and heightened susceptibility to ventricular arrhythmias. Therefore, restoring serum potassium and magnesium to optimal levels is a priority in mitigating arrhythmogenic risk (8).

Equally important is the systematic review of the patient's medication regimen to identify agents with known QT-prolonging potential. When clinically feasible, these medications should be discontinued or substituted with safer alternatives to reduce the cumulative risk of torsades de pointes and other life-threatening arrhythmias (9).

A thorough understanding of the pathophysiologic mechanisms linking impaired cardiac repolarization to ventricular electrical instability is essential for clinicians operating within the cardio-oncology domain. This knowledge enables proactive risk stratification and tailored interventions, especially in oncology patients who often present with complex pharmacologic profiles and heightened vulnerability to cardiotoxic effects.

## 4 Ventricular action potential

Understanding the ventricular action potential (VAP) is fundamental to interpret drug-induced QT prolongation and other electrophysiologic perturbations that predispose to malignant arrythmias. The VAP underlies the rhythmic depolarization-repolarization sequence that governs myocardial excitability and coordinated contraction, serving as the electrophysiological substrate upon which pharmacologic and genetic influences act (10).

**Figure 3:**
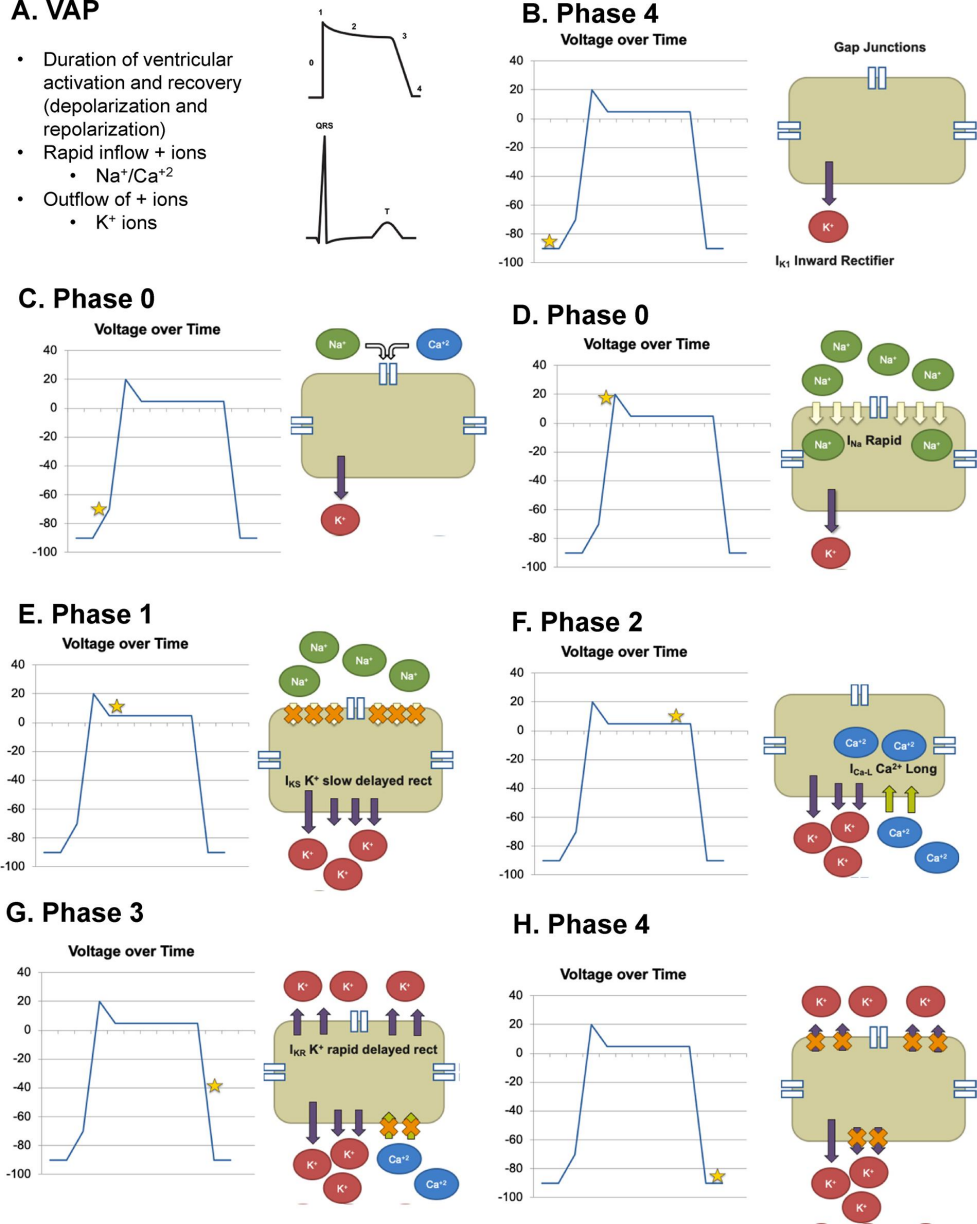
Ventricular action potential (VAP) and ionic currents across phases. **(A)** Ventricular Action Potential (VAP): Schematic representation of the duration of ventricular activation and recovery, including depolarization and repolarization. Key ionic movements include rapid inflow of Na + and Ca2 + and outflow of K + ions. **(B–H)** Phases of VAP with Voltage-Time Relationship and Major Ionic Currents: **(B)** Phase 4 (Resting Membrane Potential): Stable resting potential maintained by inward rectifier K + current (Ik1) and gap junctions. **(C,D)** Phase 0 (Rapid Depolarization): Sudden increase in membrane potential due to rapid Na + influx through fast Na + channels; minor contribution from Ca2 + entry. **(E)** Phase 1 (Initial Repolarization): Transient outward K + current (IKS) and slow delayed rectifier K + channels initiate early repolarization. **(F)** Phase 2 (Plateau): Balance between inward Ca2 + current (ICa-L) and outward K + currents maintains prolonged depolarization. **(G)** Phase 3 (Rapid Repolarization): Dominated by activation of rapid delayed rectifier K + channels and closure of Ca2 + channels, restoring negative potential. **(H)** Phase 4 (Return to Resting State): Membrane potential returns to baseline with K + efflux through inward rectifier channels and Na+/K + pump activity.

The VAP is classically divided into five sequential phases (0–4), each defined by the dominant ionic currents across the cardiomyocyte membrane (Figure 3). Phase 0, the rapid depolarization phase, is mediated by a sharp influx of sodium ions (Na^+^) through voltage-gated sodium channels (Nav1.5), generating the steep upstroke of the action potential. Phase 1 corresponds to rapid repolarization with the closing of the sodium channels and opening of transient outward potassium currents (Ito). Phase 2, the plateau phase reflects a critical equilibrium between inward L-type calcium currents (ICa,L) and outward slow delayed rectifier potassium currents (IKs), maintaining electrical stability and enabling excitation–contraction coupling. Phase 3 represents final repolarization, governed predominantly by K^+^ efflux through both rapid IKr and slow IKs channels, restoring the negative resting potential. Phase 4, the resting phase, is stabilized by the inward rectifier potassium current (IK1), which maintains the diastolic membrane potential close to the equilibrium potential for K^+^ (11).

Perturbations that reduce outward K^+^ currents (particularly IKr blockade through hERG inhibition) or enhance inward Na^+^ or Ca^2+^ currents prolong the action potential duration (APD) and, consequently, the QT interval. Such alterations, frequently induced by chemotherapeutic or supportive agents, diminish repolarization reserve and predispose to early afterdepolarizations and torsades de pointes (12).

## 5 Measuring QT interval

**Figure 4:**
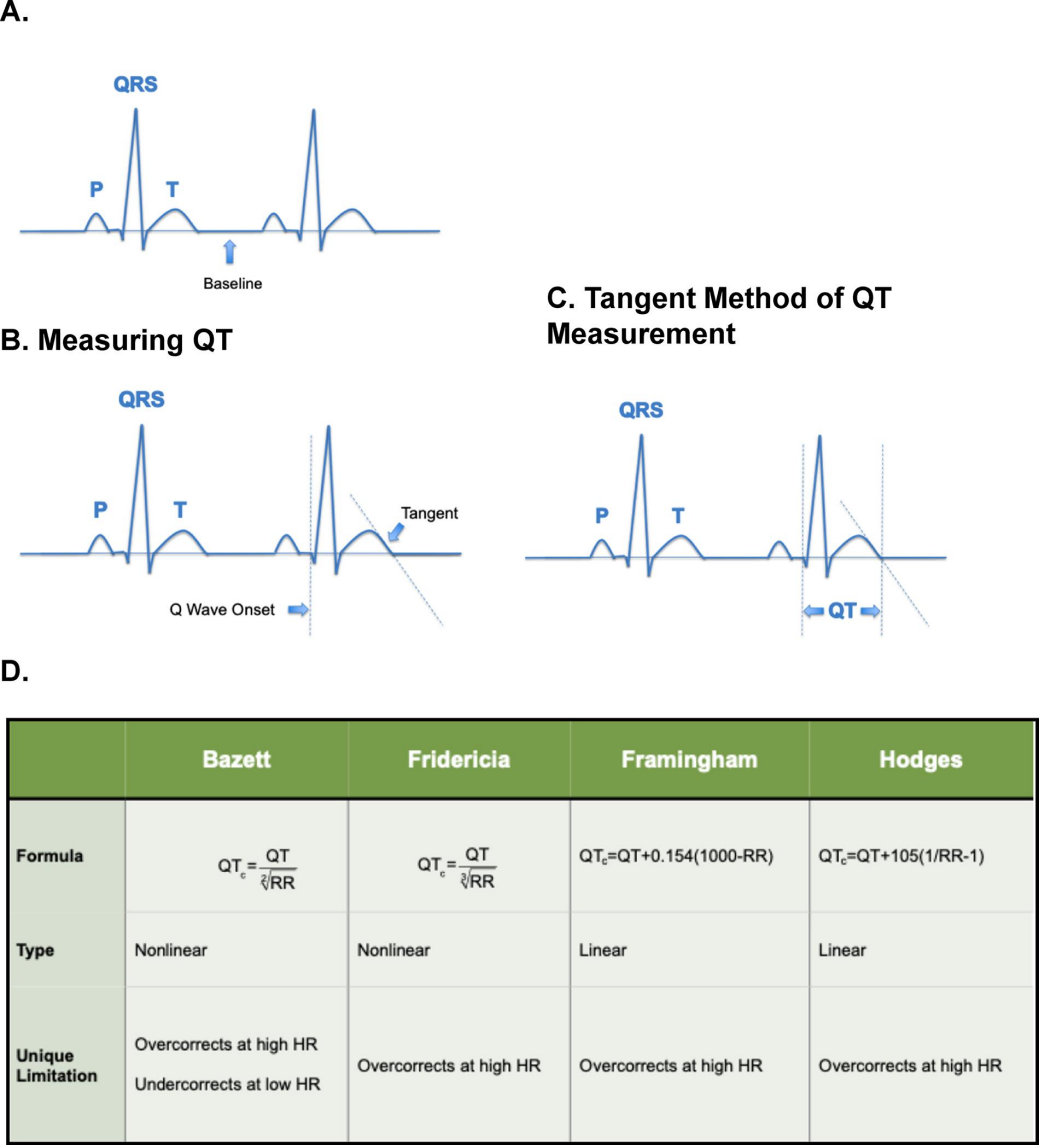
QT interval measurement and correction methods. **(A)** Normal ECG waveform illustrating P, QRS, and T waves with baseline reference. **(B)** Standard QT measurement from the onset of the Q wave to the end of the T wave. **(C)** Tangent method for QT measurement, where the end of the T wave is determined by the intersection of the tangent to the steepest slope of the terminal T wave and the baseline. **(D)** Comparison of commonly used QT correction formulas: Bazett, Fridericia, Framingham, and Hodges. Each formula is shown with its mathematical expression, classification as linear or nonlinear, and unique limitations, including overcorrection at high heart rates and undercorrection at low heart rates.

Ideal electrocardiogram (ECG) representations of QT intervals are difficult to obtain because many patients have flat T waves, which are challenging to identify. However, simple clinical methods exist that aid in measuring the QT interval. The tangent method of QT interval measurement is a simple and accurate technique that relies on recognizing key landmarks on the ECG. First, locate at least a couple of beats with similar lengths to measure. Ideally, all waves should have easily detectable T waves. Identify the isoelectric starting baseline and pinpoint the exact location of Q wave onset and draw a vertical line where the Q wave starts. Pinpointing the Q wave onset as accurately as possible is crucial for correct measurement. Then after locating the T wave, draw the steepest tangent line from the descent, and draw a vertical line to identify where the tangent line intersects with the isoelectric baseline. The QT interval is the duration between the two vertical lines (Figure 4) This straightforward method allows a quick and accurate way of measuring QT intervals in ECGs. Certain challenges still exist in measuring the QT interval, namely when the T wave is low-amplitude, biphasic, or has a U wave. Ideally ECG leads II or V5 should be used to measure the QT interval. In cases of low-amplitude ECGs, finding the lead with the clearest and longest QT interval should be used for measurement. In biphasic T waves, the tangent should be measured at the final return to baseline. And when a U wave is present, it should be excluded from the T wave and not be part of the QT measurement. Irregular rhythms such as atrial fibrillation or frequent premature complexes can also pose a challenge in accurately measuring the QT interval. In these cases, the clinician may find multiple consecutive beats with similar durations should be used to best represent the QT interval. These measurements can be averaged to find the QT.

## 6 QT syndromes and risk factors

Prolonged QT syndromes are commonly found in many cardio-oncologic patients for different reasons, for example, concurrent use of QT prolonging agents in oncology patients. Prolonged QT is either acquired or inherited and causes delays in depolarization, repolarization, or both in ventricles. This delay can predispose patients to early after-depolarizations (EADs) and manifest in commonly observed arrythmias such as Torsades de Pointes (TdP). For this reason, the Food and Drug Administration (FDA) requires most pharmaceutical trials to monitor QT prolongation during drug development. Important risk factors for both acquired and inherited QT prolongation include old age, female gender, history of myocardial ischemia, left ventricular hypertrophy, heart failure, hypothyroidism, electrolyte abnormalities, bradycardia, renal/hepatic abnormalities, concurrent use of QT prolonging agents and ion channel genetic abnormalities. Genetic or inherited prolonged QT syndromes can be divided into those that cause purely/mainly cardiac conditions and those that cause multisystemic conditions. The most common, purely cardiac conditions are the isolated long QT syndromes (LQTS), among which are three main types: Long QT type 1 (LQT1), Long QT type 2 (LQT2), and Long QT type 3 (LQT3). Multisystemic QT prolongation syndromes include Jervell and Lange-Nielsen and Andersen-Tawil syndrome (Table 1**)**. Clinically, if a patient is not on QT-prolonging medications and has normal electrolyte levels but still shows QT prolongation on an ECG, genetic testing should be considered for the early detection and treatment of these syndromes.

**Table 1:** Prolonged QT syndromes.

Syndrome	Gene Affected	Key features
Purely or Mainly Cardiac		
LQT1	KCNQ1 loss of function	- Triggered by exercise - Affected channel: Slow K channel - Most common LQTS (∼20–30%)
LQT2	KCNH2 loss of function	- Triggered by startle - Affected channel: hERG-rapid K channel - Notched T waves can be present on ECG
LQT3	SCN5A gain of function	- Triggered by sleep - Affected channel: Sodium channel problem - Autosomal dominant
Multisystemic		
Jervell & Lange-Nielsen	KCNQ1/KCNE1 loss of function (LQT1 genes)	- Autosomal recessive - Phenotype: deafness+LQTS
Andersen-Tawil	KCNJ2 loss of function	- Autosomal dominant - Phenotype: paralysis+QT changes+Epilepsy type features - Distinct facial features: Broad forehead and nasal bridge, hypertelorism, low set ears, micrognathia - Clinodactyly of 5th finger - Syndactyly of 2nd and 3rd toe

## 7 QT heart rate correction formulas

Several formulas have been developed to correct QT heart rate. The most widely used formulas are Bazett's, Fredericia's, Framingham's and Hodges's formula. These formulas involve a different set of calculations using QT and RR intervals to find the most accurate, corrected QT interval (QTc). A study by Luo and colleagues compared the effectiveness of each formula at different heart rates. In their graph that plots heart rate (BPM) vs. QT/QTc interval (ms), the more accurate formulas are represented by a more horizontal line since this translates to less QTc variations at a set heart rate (13). For example, the Bazett's formula, which calculates QTc by dividing the QT interval by the square root of the RR interval, was found to have inaccurate QTc measurements at both extremes of patient heart rates. Specifically, QTc values were either exceedingly elevated or decreased at high and low heart rates, respectively. Although none of the formulas compared were near perfect since they all possess variations at different heart rates, the Bazett's formula was the only one inaccurate at both “heart rate extremes.” As a result, Bazett's formula is represented by the least horizontal line. A shared trait among all the formulas is the tendency to elevate or overcorrect QTc values at high heart rates with only Bazett's showing under-corrected values at low heart rates. For clinical purposes, Fredericia's formula is recommended by both the Department of Health and Human Services and the FDA and is the QTc formula of choice employed at MD Anderson as well as most other hospitals (Figure 4D).

## 8 Intraventricular conduction delays and QT interval correction

Intraventricular conduction delays (IVCDs), such as Right Branch Bundle Block or Left Branch Bundle block pose a challenge in the accurate assessment of the QT interval. The standard correction formulas, including Bazett's (QTcB) and Fridericia's (QTcF) are often mistakenly applied in this context; however, because the QRS complex contributes to the QT interval, these methods can result in a falsely prolonged QT. Accurate evaluation of QT prolongation in ventricular conduction defects therefore requires adjustment for QRS duration, which can be achieved either by incorporating QRS and RR intervals as covariates in the correction formula, calculating the JT interval (QT−QRS), or the Bogossian formula (14). The JT interval offers a practical approach for QT correction in IVCD, as it is measured by subtracting the QRS duration from the QTc. Once corrected for heart rate, the primary distinction with standard QTc values lies in the reference range, which is lower than that of standard JTc (<360–370 ms). As an alternative, the QT (RR, QRS) formula which is recommended by the AHA, ACCF, and HRS ECG guidelines. Although slightly more complex than the JT interval, QTcR can be automated, incorporates both QRS and RR intervals as covariates and uses the same reference range as QTcB and QTcF. The formula is expressed as: QT RR,QRS = QT-155 × (60/HR-1) – 0.93 × (QRS – 139) + k; k −22 for men and −34 for women (14, 15). An alternative method of correction for intraventricular conduction delays such as LBBB include the Bogossian formula which is the QT interval minus 50% of the LBBB duration. This method has also been validated patients with paced rhythms (Bogossian et al. Clinical Res Cardiology Nov 2018).

## 9 Managing QTc prolongation from drug-induced risk to baseline vulnerability

The management of QT prolongation is a critical component of patient safety in cardio-oncology. The clinical complexities of this issue are best understood by examining individual patient cases, the pharmacological landscape of QT-prolonging agents, and the baseline cardiovascular profile of the cancer patient population.

A second clinical vignette effectively illustrates this challenge. The case involves a 62-year-old female with metastatic renal cell carcinoma and a pre-existing non-ischemic cardiomyopathy with a left ventricular ejection fraction of 45%–50%. Her treatment course was complex. She had previously discontinued sunitinib due to significant toxicities (pancreatitis, congestive heart failure, and acute renal failure) before progressing on an everolimus-based regimen. This latter therapy was complicated by both disease progression and significant proteinuria. Following the initiation of pazopanib, a tyrosine kinase inhibitor, she presented with a significantly prolonged QTc interval of 510 ms on her electrocardiogram. A concurrent low magnesium level of 1.7 mg/dL was also identified, a well-known contributor to delayed ventricular repolarization. The clinical management, which included discontinuing the suspected offending agent and correcting her electrolyte imbalance, resulted in a notable improvement, with the QTc interval shortening to 467 ms within five days. This case underscores the principle of reversible risk factor management and the direct impact of certain cancer therapies on cardiac repolarization.

The experience with pazopanib in this patient is representative of a broader issue, as numerous chemotherapeutic agents are implicated in QTc prolongation. The risk spans a wide range of drug classes, including antimetabolites (capecitabine), anthracyclines (epirubicin), and antimicrotubule agents (paclitaxel). The list grows substantially with targeted therapies, such as tyrosine kinase inhibitors (e.g., bosutinib, dasatinib, nilotinib, pazopanib, sunitinib), histone deacetylase inhibitors (e.g., panobinostat, romidepsin, vorinostat), proteasome inhibitors (bortezomib), CDK 4/6 inhibitors (ribociclib), B-Raf inhibitors (vemurafenib), and arsenic trioxide (16). The prevalence of these agents in modern oncology therefore calls for close cardiovascular monitoring.

Furthermore, compelling evidence suggests that cancer patients as a cohort have a different baseline cardiovascular risk profile compared to the general population. Studies comparing QTc intervals between cancer patients and non-cancer controls have demonstrated that the cancer population exhibits a rightward shift in the QTc distribution curve, indicating a tendency toward longer baseline intervals. Analysis of histograms shows this disparity clearly: using the Bazett formula, the mean QTc for cancer patients was 427 ms compared to 413 ms for controls, with the 99th percentile upper limit at 491 ms vs. 468 ms, respectively. A similar trend was observed with the Fridericia formula, which showed a mean QTc of 414 ms in cancer patients vs. 407 ms in controls, and a 99th percentile threshold of 473 ms vs. 458 ms (17). This inherent vulnerability, compounded by electrolyte disturbances and treatment with cardiotoxic agents, underscores the need for a standardized approach to cardiac monitoring in oncology to mitigate the risk of life-threatening arrhythmias.

## 10 MD Anderson's practices: QT monitoring during chemotherapy

Although there is no consensus on universal QT monitoring, the general steps that are taken for the management of QT monitoring while in MD Anderson are helpful to guide practice in cancer patients. The first step taken is to identify patients who are involved in high-risk chemotherapies (including depsipeptide, vorinostat, lapatinib, dasatinib, sunitinib, and pazopanib). These medications are known by oncologists to increase the QT interval, so they are generally referred to MD Anderson's facilities for further work-up, which generally begins with measurement of baseline electrolytes (particularly potassium and magnesium) and baseline ECG. The FDA has guidelines set in place for specific chemotherapies, which are followed by MD Anderson. These chemotherapies cannot be started in patients with congenital long QT syndrome, history of Torsade de pointes, bradyarrhythmias, or uncompensated heart failure.

**Figure 5:**
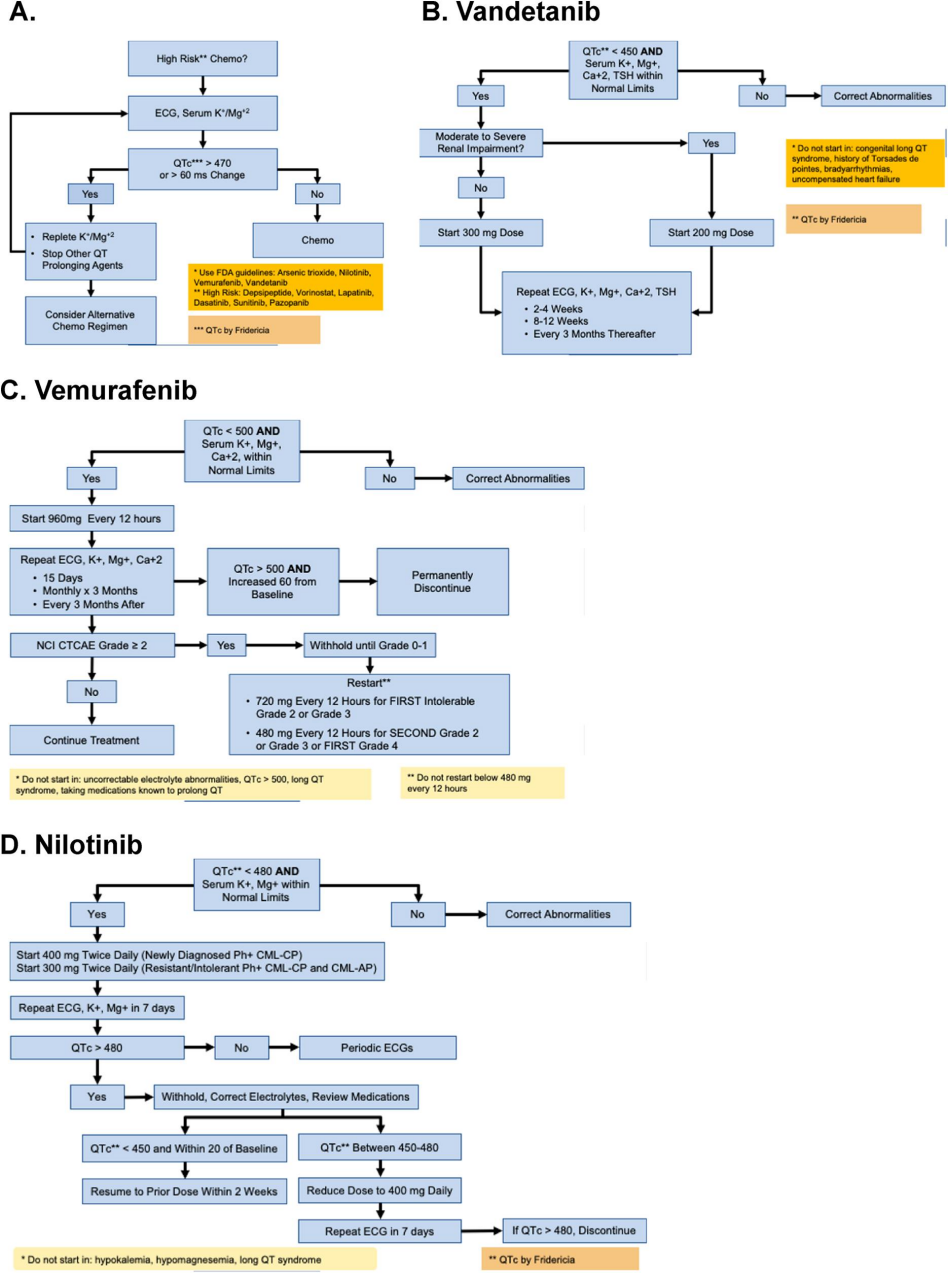
QTc monitoring and management algorithms for tyrosine kinase inhibitors. Flowcharts outlining recommended steps for QT interval monitoring and management in patients receiving QT-prolonging cancer therapies. **(A)** General approach for high-risk chemotherapy, including baseline ECG, serum potassium, and magnesium assessment, with correction of abnormalities prior to treatment initiation. **(B)** Vandetanib protocol: start at 300 mg daily if QTc <450 ms and electrolytes are normal; repeat ECG and labs at 2–4 weeks and every 3 months; discontinue for persistent QTc >500 ms. **(C)** Vemurafenib protocol: initiate 960 mg twice daily if QTc <500 ms; monitor ECG and electrolytes at baseline, after 15 days, and monthly; withhold or discontinue for QTc >500 ms or significant increases from baseline. **(D)** Nilotinib protocol: start 400 mg twice daily if QTc <480 ms; monitor ECG at baseline, day 7, and periodically; withhold or reduce dose for QTc prolongation, and discontinue if QTc ≥480 ms persists.

In patients who present with a baseline QTc exceeding 470 ms—calculated using the Fridericia formula—or who experience a QTc increase greater than 60 ms following the initiation of chemotherapy, the recommended clinical response is twofold: first, promptly correct any underlying electrolyte abnormalities; second, discontinue the implicated chemotherapeutic agent to mitigate further cardiac risk. An alternative chemotherapy regimen can be considered if the QTc prolongation or other signs such as PVCs continue to be registered even after taking the mentioned measures. If there was no initial prolongation on QTc or an increase after starting the therapy, serial ECG and close monitoring suffice for continuing the chemotherapy (Figure 5A).

### 10.1 QT monitoring during vandetanib

Vandetanib is a targeted oral therapy approved for the treatment of symptomatic or progressive medullary thyroid carcinoma (MTC) that is locally advanced or metastatic. It functions as a multi-kinase inhibitor, blocking several key signaling pathways involved in tumor growth and angiogenesis, including RET (rearranged during transfection), EGFR (epidermal growth factor receptor), and VEGFR (vascular endothelial growth factor receptor). By inhibiting these receptors, vandetanib slows tumor progression and reduces vascular supply to the cancer cells. Its use is generally reserved for patients who are not candidates for surgical resection, and it has shown efficacy in prolonging progression-free survival in this population (18).

For vandetanib, the FDA guidelines apply for patients with QTc <450 ms by Fridericia AND serum potassium, magnesium, and TSH within normal limits. If the patients' tests are not in line with these parameters, appropriate corrections must be made before starting this treatment. In patients that do fulfill those criteria, renal function must be evaluated. If there is no impairment in the renal function, patients can start with a full vandetanib dose of 300 mg. Patients with moderate-severe renal impairment can start at a lower dose of 200 mg. Once the therapy is started, close monitoring and repeat ECG, serum electrolytes, and TSH are recommended every 2–4 weeks, then spaced out to every 8–12 weeks and, if still normal, every 3 months thereafter (Figure 5B).

### 10.2 QT monitoring during vemurafenib

Vemurafenib is a selective BRAF inhibitor approved for the treatment of unresectable or metastatic melanoma harboring the BRAF V600E mutation (19). By targeting the mutated BRAF kinase within the MAPK/ERK signaling pathway, vemurafenib disrupts abnormal cell proliferation and promotes tumor regression. Its introduction marked a significant advancement in precision oncology, offering improved progression-free and overall survival for patients with BRAF-mutant melanoma. However, its use requires careful patient selection and monitoring due to a range of potential adverse effects, including dermatologic toxicity, arthralgia, and photosensitivity.

MD Anderson also follows the published FDA guidelines for QT monitoring during vemurafenib. Similarly to vandetanib, this medication is contraindicated in patients with congenital long QT syndrome, as well as patients with uncorrectable electrolyte abnormalities, QTc >500 ms or that are currently taking any medication known to prolong QT. This medication can be used in patients with QTc <500 ms by Friediricia AND have serum potassium, magnesium and calcium within normal levels. QT or electrolyte abnormalities must be corrected before starting this treatment if not within normal limits. Patients that do fulfill this criteria can start at a dose of 960 mg every 12 h. Once the medication is started, the QT interval must be followed closely by repeat ECG every 15 days for around two months, followed by monthy ECGs for 3 months, and every 3 months thereafter. If any of these ECGs show a QTc >500 ms AND a baseline increase of >60, the medication must be permanently discontinued. Following this evaluation, the patient's response to medication can be classified according to the National Cancer Institute's (NCI) Common Terminology Criteria for Adverse Events (CTCAE) (Table 2). If it is classified as a grade 0–1, the medication can be continued, but if it is classified as grade 2 or above, it is recommended by the FDA that the medication is withheld until grade 0–1 is reached. Once this grading is reached, the medication can be restarted at a lower dose of 720 mg every 12 h and continued unless the patient has an intolerable grade 2 or 3 reaction, at which the dosing must be reduced again to 480 mg every 12 h, and again must be stopped if the patient has a second grade 2 or 3 reaction or if the patient presents a grade 4 reaction (Figure 5C).

**Table 2:** National cancer institute’s (NCI) classification of prolonged corrected QT interval (QTc).

Grade	Criteria
Grade I	QTc 450–480 ms
Grade II	QTc 481–500 ms
Grade III	QTc >= 501 ms on at least two separate ECGs
Grade IV	QTc >= 501 ms or >60 ms change from baseline and TdP or polymorphic VT or signs/symptoms or serious arrhythmia
Grade V	Death

### 10.3 QT monitoring during nilotinib

Nilotinib is an FDA-approved second-generation tyrosine kinase inhibitor used to treat Philadelphia chromosome-positive chronic myelogenous leukemia (Ph + CML). While effective, it poses a risk of QT interval prolongation and potentially torsades de pointes (TdP), making it contraindicated in patients with hypokalemia, hypomagnesemia, or congenital long QT syndrome. Before initiating therapy, the QTc (Fridericia correction) must be under 480 ms and serum potassium and magnesium levels must be normal. After one week of treatment, ECG and electrolyte levels should be reassessed. If QTc exceeds 480 ms, therapy should be paused and contributing factors corrected. To resume treatment at the original dose, QTc must be below 450 ms and at least 20 ms lower than baseline. If QTc remains between 450 and 480 ms, dosing should be reduced to once daily with follow-up ECG in one week; if QTc still exceeds 480 ms, nilotinib should be discontinued (Figure 5D).

### 10.4 QT monitoring during arsenic

**Figure 6:**
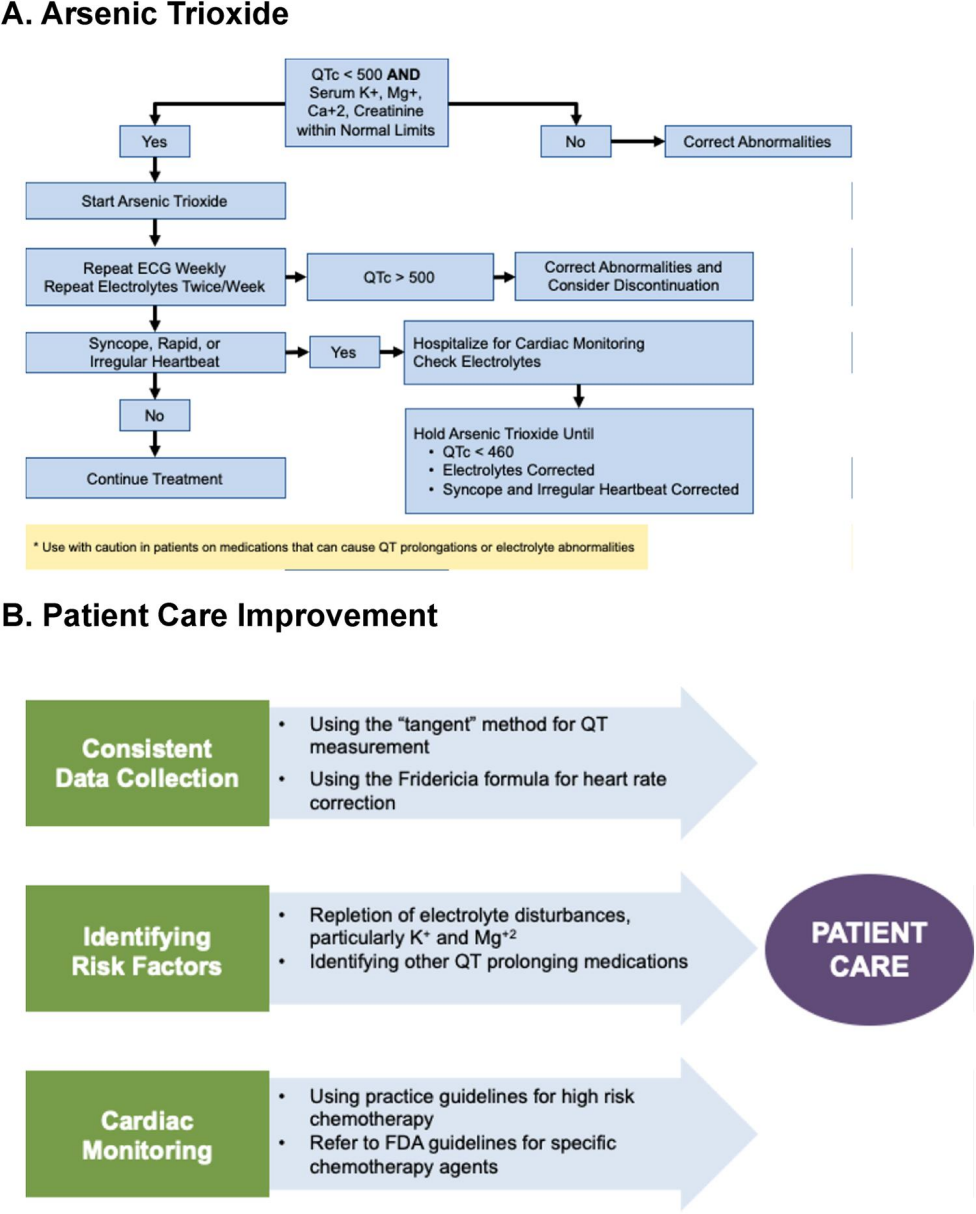
QTc monitoring and patient care strategies for arsenic trioxide therapy. **(A)** Algorithm for initiating and monitoring arsenic trioxide treatment based on QTc interval and electrolyte status. Therapy begins if QTc <500 ms and electrolytes are normal, with weekly ECG and twice-weekly electrolyte checks. QTc >500 ms or symptomatic arrhythmia prompts correction of abnormalities, hospitalization for cardiac monitoring, and temporary drug hold until QTc <460 ms and symptoms resolve.**(B)** Patient care improvement strategies to reduce QT-related risk: consistent data collection using the tangent method and Fridericia formula for QT correction; identification and correction of electrolyte disturbances (K+, Mg2+) and avoidance of other QT-prolonging drugs; and adherence to cardiac monitoring guidelines for high-risk chemotherapy per FDA recommendations.

Arsenic trioxide is an FDA-approved chemotherapeutic agent primarily used in the treatment of acute promyelocytic leukemia (APL)**,** a subtype of acute myeloid leukemia characterized by the PML-RAR*α* fusion gene (20, 21). It induces apoptosis and promotes differentiation of leukemic cells, contributing to remission in APL patients. While effective, it is associated with QT interval prolongation, which can lead to TdP or other life-threatening arrhythmias in a subset of patients. The FDA recommends a QTc threshold of less than 500 ms for initiating therapy—higher than that required for other high-risk agents. Once treatment begins, patients should undergo weekly ECGs and electrolyte monitoring. If the QTc exceeds 500 ms, clinicians should correct any electrolyte abnormalities and consider discontinuing arsenic. Symptomatic patients must have treatment held and be hospitalized for cardiac monitoring and expedited evaluation. Therapy should only be resumed once symptoms have resolved, electrolytes are normalized, and the QTc has decreased to below 460 ms, a threshold significantly lower than the initial requirement for treatment initiation (Figure 6A).

## 11 Conclusion

In summary, effective management of QT interval prolongation in oncology requires a structured, multi-pronged approach centered on consistent methodology, proactive risk stratification, and vigilant cardiac surveillance. Standardizing QT interval measurement—particularly through manual use of the tangent method and correction via the Fridericia formula—ensures reproducibility and accuracy across patient populations and clinical settings. Identifying patients with predisposing factors such as electrolyte imbalances, underlying cardiac conditions, or concurrent use of QT-prolonging medications allows for timely intervention and tailored monitoring strategies. Patient education plays a critical role in early detection; counseling individuals on symptoms such as palpitations, dizziness, or syncope can prompt earlier reporting and evaluation. Given the extensive and evolving list of medications associated with QT prolongation, clinicians are encouraged to utilize resources like https://crediblemeds.org, which offers an up-to-date database of implicated drugs. For high-risk chemotherapeutic agents, institutional protocols that define thresholds for treatment initiation, dose adjustment, and monitoring frequency are essential—particularly when formal guidelines are lacking. Establishing these internal standards promotes consistency, enhances patient safety, and supports evidence-based decision-making in cardio-oncology practice (Figure 6B).

## References

[1] Chohan PS Mittal R Javed A. Antipsychotic medication and QT prolongation. Pak J Med Sci. (2015) 31:1269–71. 10.12669/pjms.315.8998 26649027 PMC4641296

[2] Wisniowska B Tylutki Z Wyszogrodzka G Polak S. Drug-drug interactions and QT prolongation as a commonly assessed cardiac effect—comprehensive overview of clinical trials. BMC Pharmacol Toxicol. (2016) 17:12. 10.1186/s40360-016-0053-1 26960809 PMC4785617

[3] Kloth JS Pagani A Verboom MC Malovini A Napolitano C Kruit WH Incidence and relevance of QTc-interval prolongation caused by tyrosine kinase inhibitors. Br J Cancer. (2015) 112:1011–6. 10.1038/bjc.2015.82 25742483 PMC4366905

[4] Barbey JT Pezzullo JC Soignet SL. Effect of arsenic trioxide on QT interval in patients with advanced malignancies. J Clin Oncol. (2003) 21:3609–15. 10.1200/JCO.2003.10.009 14512391

[5] Gavioli EM Guardado N Haniff F Deiab N Vider E. The risk of QTc prolongation with antiemetics in the palliative care setting: a narrative review. J Pain Palliat Care Pharmacother. (2021) 35:125–35. 10.1080/15360288.2021.1900491 33974499

[6] Sauer AJ Newton-Cheh C. Clinical and genetic determinants of torsade de pointes risk. Circulation. (2012) 125:1684–94. 10.1161/CIRCULATIONAHA.111.080887 22474311 PMC3347483

[7] Drew BJ Ackerman MJ Funk M Gibler WB Kligfield P Menon V Prevention of torsade de pointes in hospital settings: a scientific statement from the American Heart Association and the American College of Cardiology foundation. J Am Coll Cardiol. (2010) 55:934–47. 10.1016/j.jacc.2010.01.001 20185054 PMC3057430

[8] Khatib R Sabir FRN Omari C Pepper C Tayebjee MH. Managing drug-induced QT prolongation in clinical practice. Postgrad Med J. (2021) 97:452–8. 10.1136/postgradmedj-2020-138661 33122341 PMC8237186

[9] Thomas SH Behr ER. Pharmacological treatment of acquired QT prolongation and torsades de pointes. Br J Clin Pharmacol. (2016) 81:420–7. 10.1111/bcp.12726 26183037 PMC4767204

[10] Wei X Yohannan S Richards JR. Physiology, Cardiac Repolarization Dispersion and Reserve. Treasure Island (FL): StatPearls (2025). 30725879

[11] Joukar S. A comparative review on heart ion channels, action potentials and electrocardiogram in rodents and human: extrapolation of experimental insights to clinic. Lab Anim Res. (2021) 37:25. 10.1186/s42826-021-00102-3 34496976 PMC8424989

[12] Yap YG Camm AJ. Drug induced QT prolongation and torsades de pointes. Heart. (2003) 89:1363–72. 10.1136/heart.89.11.1363 14594906 PMC1767957

[13] Luo S Michler K Johnston P Macfarlane PW. A comparison of commonly used QT correction formulae: the effect of heart rate on the QTc of normal ECGs. J Electrocardiol. (2004) 37(Suppl):81–90. 10.1016/j.jelectrocard.2004.08.030 15534815

[14] Rautaharju PM Zhang ZM Prineas R Heiss G. Assessment of prolonged QT and JT intervals in ventricular conduction defects. Am J Cardiol. (2004) 93:1017–21. 10.1016/j.amjcard.2003.12.055 15081446

[15] Rautaharju PM Surawicz B Gettes LS Bailey JJ Childers R Deal BJ AHA/ACCF/HRS recommendations for the standardization and interpretation of the electrocardiogram: part IV: the ST segment, T and U waves, and the QT interval: a scientific statement from the American Heart Association electrocardiography and arrhythmias committee, council on clinical cardiology; the American College of Cardiology foundation; and the heart rhythm society. Endorsed by the international society for computerized electrocardiology. J Am Coll Cardiol. (2009) 53:982–91. 10.1016/j.jacc.2008.12.014 19281931

[16] Kim PY Irizarry-Caro JA Ramesh T Iliescu C Lopez-Mattei JC. How to diagnose and manage QT prolongation in cancer patients. JACC CardioOncol. (2021b) 3:145–9. 10.1016/j.jaccao.2021.01.002 34396315 PMC8352274

[17] Kim P Masha L Olson A Iliescu C Karimzad K Hassan S QT Prolongation in cancer patients. Front Cardiovasc Med. (2021a) 8:613625. 10.3389/fcvm.2021.613625 33718445 PMC7946823

[18] Ton GN Banaszynski ME Kolesar JM. Vandetanib: a novel targeted therapy for the treatment of metastatic or locally advanced medullary thyroid cancer. Am J Health Syst Pharm. (2013) 70:849–55. 10.2146/ajhp120253 23640345

[19] Corrie PG Terheyden P Ten Tije AJ Herbst R Jansen R Marples M A prospective observational safety study of patients with BRAF(V) (600) -mutated unresectable or metastatic melanoma treated with vemurafenib (zelboraf safety study). Br J Dermatol. (2019) 180:1254–5. 10.1111/bjd.17465 30488430

[20] Zhang XW Yan XJ Zhou ZR Yang FF Wu ZY Sun HB Arsenic trioxide controls the fate of the PML-RARalpha oncoprotein by directly binding PML. Science. (2010) 328:240–3. 10.1126/science.1183424 20378816

[21] Yan M Wang H Wei R Li W. Arsenic trioxide: applications, mechanisms of action, toxicity and rescue strategies to date. Arch Pharm Res. (2024) 47:249–71. 10.1007/s12272-023-01481-y 38147202

